# Prevalence of Sleep Disorders Among Patients With Type 2 Diabetes Mellitus in Makkah City: A Cross-Sectional Study

**DOI:** 10.7759/cureus.33088

**Published:** 2022-12-29

**Authors:** Walaa M Alamer, Razan M Qutub, Esraa A Alsaloumi, Nujood K Natto, Reem M Alshehri, Abdullah Khafagy

**Affiliations:** 1 College of Medicine, Umm Al-Qura University, Makkah, SAU; 2 College of Nursing, Umm Al-Qura University, Makkah, SAU; 3 Medicine and Surgery, Umm Al-Qura University, Makkah, SAU; 4 College of Medicine, Ibn Sina National College, Jeddah, SAU; 5 Community Medicine and Pilgrims Healthcare, Umm Al-Qura University, Makkah, SAU

**Keywords:** t2dm, type 2 diabetes mellitus, pittsburgh sleep quality index, risk factor, sleep disorder, poor sleep, type 2 diabetes, prevalence

## Abstract

Background: Due to rising rates of morbidity and mortality associated with type 2 diabetes mellitus (T2DM), Saudi Arabia is ranked second in the Middle East and seventh overall among nations with the greatest incidence of diabetes mellitus (DM). Significant sleep abnormalities have been linked to difficulties in managing blood sugar levels, suggesting a link between sleep disorders and diabetes. This study aimed to find out how common sleep disturbances were among patients with T2DM in Makkah, Saudi Arabia.

Methods: This descriptive cross-sectional study was conducted between June and August 2022 in Makkah City, Saudi Arabia. Patients with T2DM who visited primary healthcare facilities in Makkah during the study's duration were included in the study. To evaluate sleep quality, the Pittsburgh Sleep Quality Index in Arabic was employed. Patients who met the inclusion criteria were given an interview questionnaire to fill out.

Results: In total, 355 patients with T2DM were enrolled in this study. The patients' median age was 49.24 years. Other than DM, a majority of them (58.9%) had chronic illnesses, with hypertension (64.5%) and cardiovascular disease (65.5%) as the most prevalent comorbidities. Only 22% of the patients had controlled diabetes. Of the individuals who had sleep disorders, 63.7% stated having poor sleep quality.

Conclusion: Sleep problems are a common occurrence in patients with T2DM. Additionally, compared to people with other chronic disorders, people with endocrine diseases had poorer sleep quality. Hence, the duration of diabetes has an impact on sleep quality.

## Introduction

Patients with type 2 diabetes mellitus (T2DM) frequently experience sleep issues. Additionally, patients with endocrine dysfunction experience lower quality of sleep compared to those with other chronic disorders. Sleep quality is impacted by prolonged diabetes duration [1-3].

Sleep disturbances have been known to cause inflammatory processes, predisposing a person to develop diabetes [4]. Uncontrolled diabetes has been linked to a reduction in sleep quality [4]. According to prior studies, more than 50% of patients with T2DM have trouble sleeping [5]. Additionally, ineffective glucose management is hampered by poor sleep, which is linked to diabetes-related complications [5].

Patients with sleep-disordered breathing may have impaired glucose metabolism due to obstructive sleep apnea (OSA). The American Academy of Sleep Medicine defines OSA as "repeated episodes of total or partial upper airway obstruction while sleeping" [1]. These complete or incomplete obstructions cause brief awakenings and a decline in blood oxygen saturation [6]. Respiratory disturbances may cause frequent arousals, which could lead to sleep loss and predispose to metabolic disorders [7]. Untreated OSA is also linked to a number of harmful health outcomes, such as chronic fatigue syndrome, decrease in quality of life, increase in the risk of death, systemic hypertension (HTN), diabetes, coronary artery disease, stroke, atrial fibrillation, and congestive heart failure [8].

In a prior study at King Abdulaziz Medical City in the Kingdom of Saudi Arabia, it was discovered that obesity with a body mass index of more than 30 kg/m2 (39.1%), HTN (33.9%), diabetes mellitus (DM) (20.8%), depression (4.3%), asthma (17.3%), chronic obstructive pulmonary disease (6.6%), and hyperlipidemia (2.7%) were the conditions most often related to a less than seven hours of sleep per night [9]. Another study conducted in Jazan found that the occurrence of poor sleep quality is 55.4% (95% CI: 49.7-60.8) and that it is related to sociodemographic and factors like being elderly, female, uneducated, smoker, with diabetes or other comorbidities, or having psychiatric symptoms [10]. According to a study done in Taif, 26.9% of patients had a moderate risk for OSA, while 15.2% of patients had a severe risk.

When combining both oral medications and insulin, more patients had a serious risk of sleep abnormality than when taking either oral medications alone or insulin alone [11]. Research on the prevalence of sleep problems in people with T2DM is scarce in Saudi Arabia. Consequently, the purpose of this study was to evaluate the prevalence of sleep difficulties among people with T2DM and its contributing factors in the Makkah region of Saudi Arabia.

## Materials and methods

Study design, duration, and setting

This was a descriptive, cross-sectional study carried out in Makkah City, Saudi Arabia from June to August 2022.

Participants

The study included patients with T2DM who visited primary healthcare facilities in the Makkah region during the study period. Patients with T2DM who were ≥18 years old and of either sex were required to meet the inclusion criteria. Patients with type 1 DM, those with known psychiatric conditions, people who take sleeping medicines, women who are pregnant, and people who work night shifts were all excluded from the study.

Sampling methodology

Patients were randomly chosen from the lists of patients in the primary healthcare facilities in the Makkah region. Recruitment was carried out at each healthcare facility until the appropriate number of participants was reached.

Determination of sample size

Using OpenEpi (version 3.0), the minimum sample size required for this investigation was calculated [12]. The number of T2DM patients [13], 95% confidence interval (CI), an anticipated frequency of 50%, and a design effect of 1 were taken into account while calculating the sample size. The calculated minimum sample size was 334 patients.

Data collection

Patients who met the inclusion criteria were given a questionnaire, which was then used to gather data. After receiving the participants' informed consent, face-to-face interviews were conducted and the questionnaires were given. The following information was gathered: demographics, the number of children, type of dwelling, employment status, smoking, income level, BMI, duration of diabetes, chronic conditions, and glycosylated hemoglobin (HbA1c) levels.

To evaluate the quality of sleep, the validated Pittsburgh Sleep Quality Index (PSQI) was utilized [14]. This scale was used to evaluate the quality of sleep over the previous month and comprises seven components on the following items: daytime dysfunction, daytime dysfunction quality, daytime dysfunction duration, and daytime dysfunction efficiency. Each element is given a score between 0 and 3, and the PSQI values range from 0 to 21. Poor sleep quality is indicated by high scores, with PSQI scores of more than 5 having a sensitivity of 89.6% and a specificity of 86.5% [14].

Data analysis

Data were statistically analyzed using SPSS version 26 software (IBM Corp., Armonk, NY). The chi-squared test (χ2) was used for analyzing qualitative data, which were expressed as percentages and numbers, and for examining the variables. Quantitative data were presented as mean and standard deviation (mean ± D). Correlation analysis was performed using Spearman's test. A p-value < 0.05 was considered statistically significant.

## Results

After non-respondents were excluded from the trial, 355 patients remained. The patients' average BMI was 28.35 kg/m2, with an average age of 49.24 years (Table 1). Each patient had an average of four children (± 1.17). The patients' mean HbA1c level was 7.22 ± 1.25, and they had had diabetes for an average of 8.83 ± 7.99 years. Males made up 58.9% of the patients, Saudi nationals made up 92.1% of the patients, and 49.9% of the patients had a university or diploma-level education. Of the patients, 36.9% lived in rented homes, and 72.1% of the patients were married. Of them, 41.4% earned between 10,000 and 20,000 Saudi riyals (SR) per month, while 24.2% of them were unemployed. Of them, 36.3% were smokers and the most common comorbidities were cardiovascular disease (CVD) (65%) and HTN (64.5%).

**Table 1:** Distribution of studied diabetic patients according to their demographics, BMI, DM duration, HbA1C level, and comorbidities (N = 355) DM: diabetes mellitus; HbA1C: glycosylated hemoglobin; SR: Saudi riyals; CVS: cardiovascular system; HTN: hypertension.

Variable	No. (%)
Age (years)	49.24 ± 16.14
BMI (kg/m^2^)	28.35 ± 3.12
Number of children	4.09 ± 1.17
DM duration (years)	8.83 ± 7.99
HbA1C level	7.22 ± 1.25
Sex	
Female	146 (41.1)
Male	209 (58.9)
Nationality	
Saudi	327 (92.1)
Non-Saudi	28 (7.9)
Educational level	
Less than secondary school	118 (33.2)
University/diploma	177 (49.9)
PhD	33 (9.3)
Master's degree	27 (7.6)
Marital status	
Widow	28 (7.9)
Single	71 (20)
Married	256 (72.1)
Employment status	
Unemployed	86 (24.2)
Retired	93 (26.2)
Employee in the private sector	69 (19.4)
Employee in the governmental sector	107 (30.1)
House	
Rented	131 (36.9)
Owned	224 (63.1)
Monthly income	
<10,000 SR	149 (42)
10,000-20,000 SR	147 (41.4)
>20,000 SR	59 (16.6)
Smoking status	
Non-smoker	226 (63.7)
Smoker	129 (36.3)
Other chronic diseases	
Yes	209 (58.9)
No	146 (41.4)
If yes, what disease? (N = 209)	
Endocrine	39 (18.4)
CVS	136 (65)
HTN	135 (64.5)
Respiratory disease	51 (24.4)
Kidney disease	20 (9.5)
Liver disease	4 (1.9)
Neuropathy and retinopathy	13 (6.2)
DM control	
Controlled	78 (22)
Uncontrolled	277 (78)

**Figure 1:**
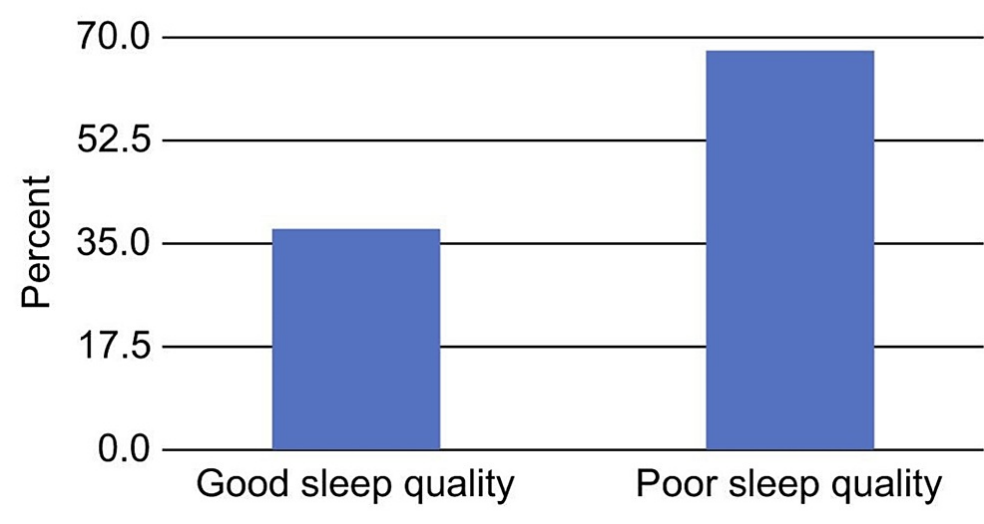
Distribution of the participants according to sleep quality based on their Pittsburgh Sleep Quality Index scores (N = 355)

The average PSQI score for patients was 7.25. After categorizing the patients' PSQI scores, it was discovered that 63.7% of them experienced poor sleep (Figure 1).

When compared to patients without endocrine disorders, patients with endocrine disorders other than DM had a substantially greater percentage of people who reported having bad sleep (p < 0.05; Table 2). Patient demographics, BMI, DM duration, HbA1c level, and other comorbidities were all connected with sleep quality (p > 0.05; Table 2).

**Table 2:** Relationship between sleep quality and patients' demographics, BMI, DM duration, HbA1C level, and comorbidities (N = 355) * Mann-Whitney test. DM: diabetes mellitus; HbA1C: glycosylated hemoglobin; CVS: cardiovascular system; HTN: hypertension.

Variable	Sleep quality		χ2	P-value
	Good, No. (%)	Poor, No. (%)		
Age	49.35 ± 15.12	49.18 ± 16.71	0.009*	0.93
BMI	28.3 ± 83.1	28.33 ± 3.14	0.71*	0.475
Number of children	4.05 ± 2.89	4.11 ± 3.33	0.13*	0.894
DM duration	7.59 ± 6.66	9.54 ± 8.59	1.54*	0.122
HbA1C level	7.18 ± 1.09	7.25 ± 1.33	0.007*	0.994
Sex				
Female	47 (32.2)	99 (67.8)	1.84	0.175
Male	82 (39.2)	127 (60.8)		
Nationality				
Saudi	121 (37)	206 (63)	0.79	0.373
Non-Saudi	8 (28.6)	20 (71.4)		
Educational level				
Less than secondary school	45 (38.1)	73 (61.9)	4.72	0.193
University/diploma	56 (31.6)	121 (68.4)		
PhD	16 (48.5)	17 (51.5)		
Master's degree	12 (44.4	15 (55.6)		
Marital status				
Widow	7 (25)	21 (75)	3.25	0.196
Single	22 (31)	49 (69)		
Married	100 (39.1)	156 (60.9)		
Employment status				
Unemployed	26 (30.2)	60 (69.8)	2.33	0.506
Retired	33 (35.5)	60 (64.5)		
Employee in the private sector	27 (39.1)	42 (60.9)		
Employee in the governmental sector	43 (40.2)	64 (59.8)		
Smoking status				
Non-smoker	80 (35.4)	146 (64.6)	0.23	0.626
Smoker	49 (38)	80 (62)		
Other chronic diseases				
Yes	71 (34)	138 (66)	1.23	0.267
No	58 (39.7)	88 (60.3)		
Type of chronic disease				
Endocrine	7 (5.4)	32 (14.2)	6.4	0.011
CVS	47 (36.4)	89 (39.4)	0.3	0.583
HTN	46 (35.7)	89 (65.9)	0.48	0.487
Respiratory disease	22 (17.1)	29 (12.8)	1.19	0.275
Kidney disease	7 (5.4)	13 (5.8)	0.01	0.898
Liver disease	2 (1.6)	2 (0.9)	0.32	0.568
Neuropathy and retinopathy	7 (5.4)	6 (2.7)	1.78	0.181
DM control				
Controlled	27 (34.6)	51 (65.4)	0.12	0.72
Uncontrolled	102 (36.8)	175 (63.2)		

A non-significant positive association was found between the PSQI score and age, the number of children, and the HbA1c level (p > 0.05; Table 3). Furthermore, a negative correlation between PSQI score and BMI (p > 0.05) was found. The outcome, nevertheless, was not noteworthy.

**Table 3:** Spearman's correlation analysis between Pittsburgh Sleep Quality Index (PSQI) scores and participants’ age, BMI, number of children, and HbA1C level HbA1C: glycosylated hemoglobin.

Variable	Pittsburgh Sleep Quality Index (PSQI) score	
	r	P-value
Age	0.002	0.968
BMI	-0.02	0.588
Number of children	0.008	0.879
HbA1C level	0.03	0.574

**Figure 2:**
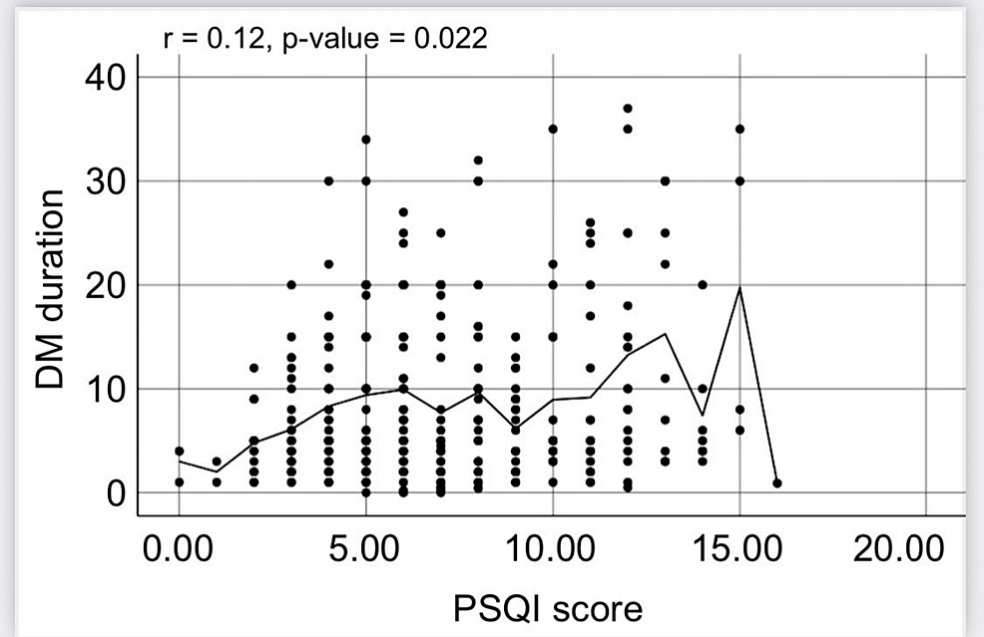
Spearman's correlation analysis of the correlation between the Pittsburgh Sleep Quality Index (PSQI) and the duration of diabetes mellitus (DM)

Additionally, DM duration and PSQI score had a strong positive correlation (r = 0.12, p = 0.022; Figure 2). The outcome, nevertheless, was not substantial.

**Figure 3:**
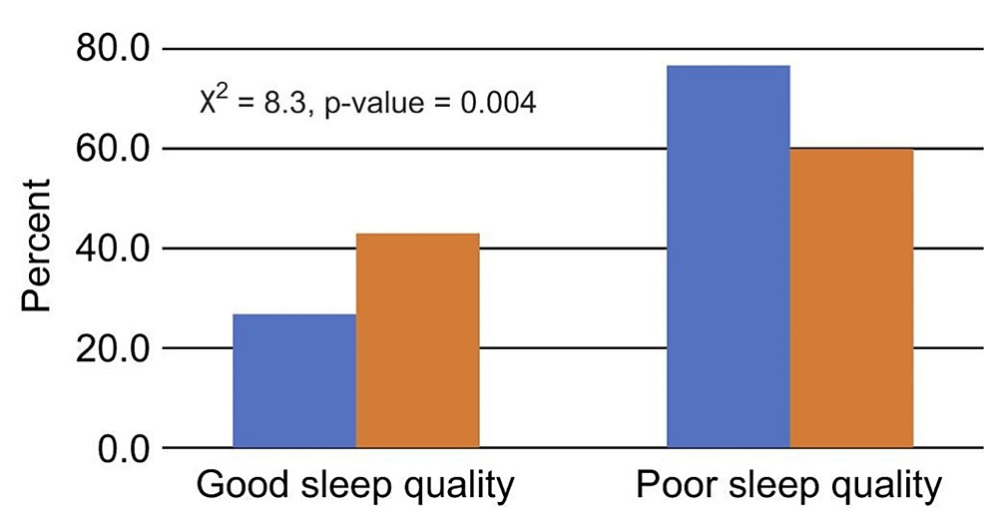
Relationship between sleep quality and type of residence

**Figure 4:**
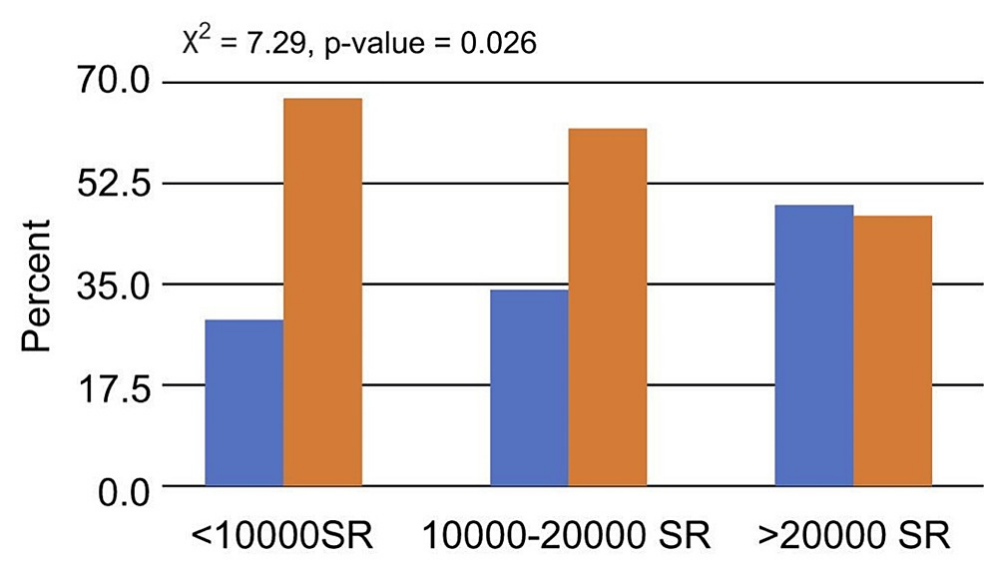
Relationship between sleep quality and monthly income

Patients who lived in rented homes or had low monthly incomes (10,000 SR) had a noticeably greater percentage of those who reported having bad sleep than patients who owned their homes or had higher monthly incomes (p < 0.05; Figures 3, 4).

## Discussion

The goal of this cross-sectional study was to determine the prevalence of sleep disorders among patients with T2DM in Makkah, Saudi Arabia. In the present study, more than half (63.75) of the patients were classified as having poor sleep quality based on their PSQI scores. Studies conducted in Jazan (55.4%) and Abha (72%) yielded similar results [15,16]. The present study revealed that the socioeconomic status and comorbidities of the patients strongly influenced the prevalence of poor sleep quality.

More than half of the patients in the current study were men with high BMI. This result is in line with a study done in Japan, where 342 participants (82.5% of whom were men) had T2DM and poor sleep quality [17]. The two comorbid conditions that affected patients with T2DM the most in the current study were CVD and HTN. This result is in line with a previous Ethiopian study, which discovered that patients with T2DM and HTN were more likely than those with T2DM alone to have poor sleep quality [18]. Moreover, the current study found that those with low earnings had worse sleep quality (42%) than people with high incomes in terms of socioeconomic characteristics. Similar results were found in a study done in Turkey using the PSQI, where 54% of the female participants and 50% of the male participants reported having poor sleep quality [7]. Poor sleep quality and type of dwelling were shown to be substantially associated in the current study. Compared to patients who lived in their own homes, those who leased homes had poorer sleep quality. However, there is not enough evidence to support this conclusion in the present.

DM is one of the most widespread diseases in the world. DM has been linked to a number of chronic illnesses, including OSA, in addition to diabetic complications such as neuropathy and neuropathic pain, which were found to disrupt sleep [1]. Additional sleep-related diabetes symptoms include polyuria, nocturia, snoring, and prolonged daytime tiredness. Diabetes and snoring are related conditions. Furthermore, chronic daytime sleepiness might cause insulin resistance [19]. Depression, cerebrovascular accidents, CVD problems, and HTN can all have a poor impact on sleep and general quality of life. Insufficient or disturbed sleep can have a detrimental influence on a patient's recovery, ability to control their diabetes, and quality of life. Healthcare professionals must address these problems to provide DM patients with the best care possible. Additionally, sleep education is a vital method for improving the sleep quality of these patients [20].

Of the participants of the current study, 63.7% were classified as having poor sleep quality based on their PSQI ratings. Additionally, the findings showed a positive correlation between PSQI score and age, HbA1c level, and the number of children (p = 0.05). Additionally, the results showed a negative correlation between BMI and PSQI score (p = 0.05). Additionally, there was a strong significant correlation between PSQI score and DM duration (r = 0.12, p = 0.022). The association between PSQI subscales and diabetes control was also supported by a different study that reached a similar conclusion [14]. The Simultaneous Risk Factor Control Using Telehealth project participants in that study completed surveys, and the findings showed that patients with HbA1c levels below 7% had significantly lower global and PSQI subscale ratings than those in the control group (p = 0.01) [14].

The findings of the current investigation are supported by the results of a separate cross-sectional study done in Turkey by Ayaz and Dincer in 2021. The study found a strong relationship between low sleep quality and high HbA1c levels in patients with T2DM [20].

Limitations

The limitations of this study are numerous. Firstly, clinical data were gathered based on patients' answers, for example, HbA1c levels. This poses a significant bias. Also, the background section outlines how anti-hyperglycemic medication and sleep quality are associated. Secondly, the projected sample size was initially not attained by the actual sample size, but statistical analysis nevertheless produced meaningful results. Thirdly, because this was a cross-sectional study, it was unable to determine how sleep disturbances and diabetes relate to one another. Moreover, the patients verbally provided their data. Hence, data on BMI and Hb1Ac levels may not have been accurate.

## Conclusions

This study demonstrated that people with T2DM frequently experience sleep difficulties. Additionally, the findings showed that patients with endocrine disorders have poorer sleep quality than individuals with other chronic diseases. The findings also indicated that prolonged hyperglycemia has an impact on sleep quality.
